# Imperfection in Semiconductors Leading to High Performance Devices

**DOI:** 10.1002/advs.202516270

**Published:** 2025-12-19

**Authors:** Jean‐Yves Duboz, Matilde Siviero, Lucas Lesourd, Eric Frayssinet, Sebastien Chenot, Petter Hofverberg, Sullivan Marafico, Marie Vidal, Maxime Hugues

**Affiliations:** ^1^ CNRS CRHEA Université Côte d'Azur Valbonne France; ^2^ Fédération Claude Lalanne Cyclotron Biomédical Centre Antoine Lacassagne Université Côte d'Azur Nice France

**Keywords:** defect, detection, semiconductors, sensitivity, transport

## Abstract

Semiconductors form the basis of high‐performance optoelectronic devices, enabling efficient light emission and detection. While crystalline perfection is generally sought to optimize device performance, specific lattice defects can endow materials with unexpected and useful functionalities. Here, we show that engineered defect states in gallium nitride (GaN) diodes markedly enhance their response to high‐energy protons. Through a combination of device simulations and experimental measurements, we demonstrate that forward biasing the diode just below its turn‐on voltage activates a defect‐mediated photoconductive regime. This operating mode induces substantial carrier trapping and photoconductive gain while simultaneously suppressing the dark current—a behaviour in stark contrast to conventional photoconductors. The exploitation of this previously underexplored detection mechanism yields a three‐orders‐of‐magnitude enhancement in sensitivity over standard photovoltaic operation, enabling reliable quantification of proton fluxes down to a few particles per second. This novel mode of operation is not limited to protons but also extends to X‐rays and other high‐energy particles, and may be generalized to a broader class of semiconductors exhibiting high levels of doping compensation. These findings open new avenues for very low‐flux particle detection across diverse application spaces, including medical, astronomy, and industrial imaging.

## Graphical abstract

Crystalline perfection is typically pursued in semiconductors to enhance device performance. However, through modeling and experimental work, we show that defects can be strategically employed in a specific detection regime to increase sensitivity to extreme values. GaN diodes are demonstrated to effectively detect high‐energy proton beams at fluxes as low as a few protons per second.

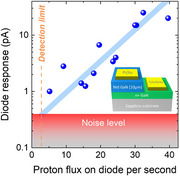

## 1 Introduction

**FIGURE 1:**
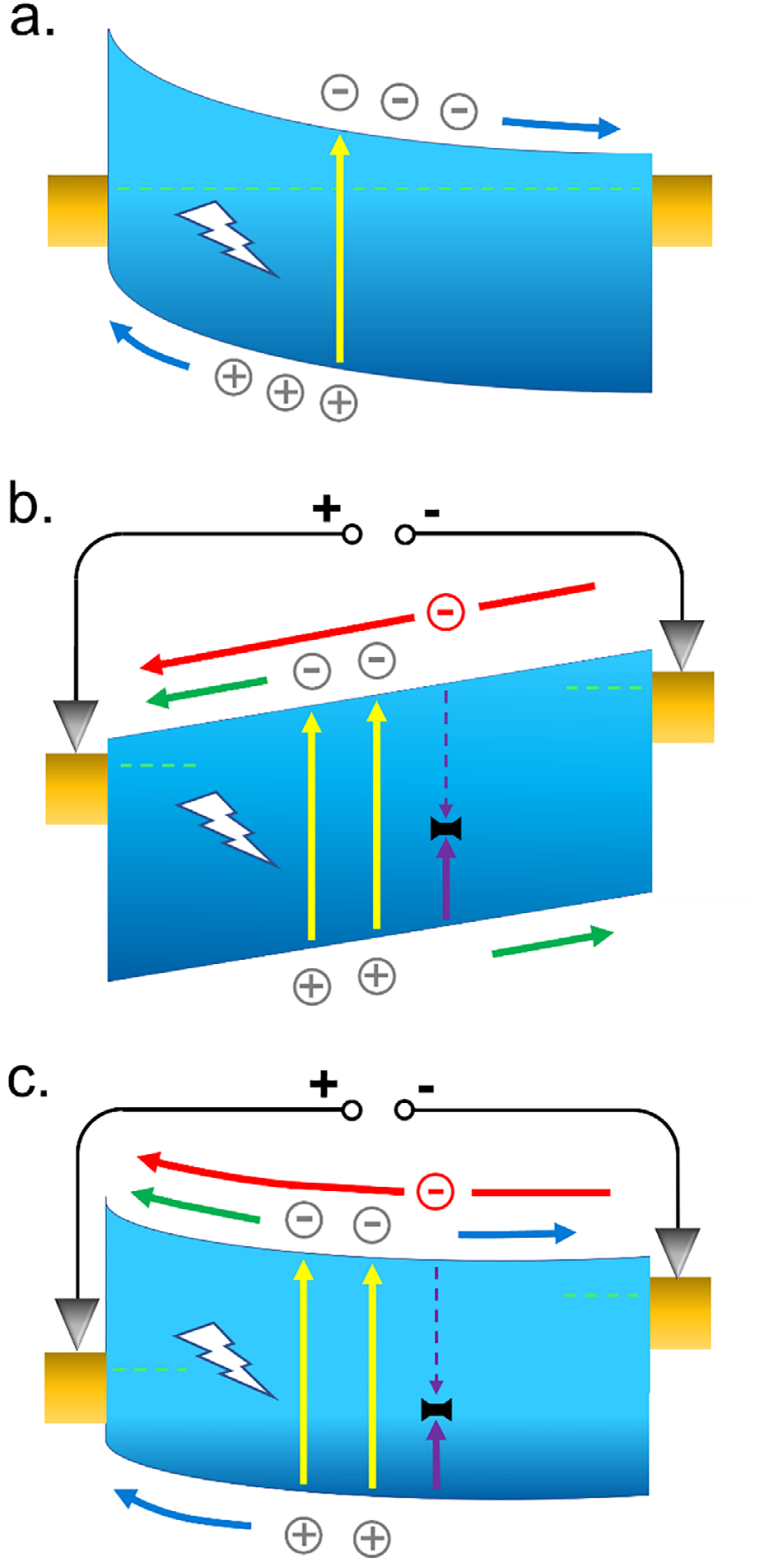
Working principle of the photodetection modes. Schematic band profiles in semiconductor detectors: (a) for a photovoltaic detector at zero bias, carriers are swept by the strong electric field with negligible recombination and a null dark current (b) for a photoconductor under bias, carriers are drifted by a weak electric field and electroneutrality is provided by carrier injection from the contacts. In the case of trapping of one type of carrier, the other carrier type circulates in the device until it recombines and restores the neutrality. The dark current is large (c) for a photodiode under small forward bias. Both processes described in a) and b) coexist. However, the dark current is limited by the remaining build‐in energy barrier, leading to a large response to dark current ratio, corresponding to a large sensitivity.

In a perfectly periodic material, Bloch's theorem states that electron wave functions can be expressed as plane waves modulated by periodic functions [1]. Semiconductor band structures can then be deduced, with the band gap as an important parameter. Transitions across the band gap can be induced by an energy exchange with photons or phonons for instance. Emblematic transitions are light emission and absorption in semiconductor, with a photon energy equal to the band gap energy. Photodetectors are fabricated based on such transitions: one absorbed photon induces the creation of an electron‐hole pair. The collection of these charges yields a photocurrent. In photovoltaic devices [2] a current is generated without external bias (see Figure 1a), while the photoconductive devices [3] require an external bias to generate a photocurrent in response to light (Figure 1b). In the best case in photovoltaic devices, charges are fully collected and the quantum efficiency is unity [4]. Photoconductive devices may present some gain due to the difference of mobility between electrons and holes [4]. It is important to note that this description only applies in perfect semiconductors, so without any defect. Materials are however never perfect and the translational symmetry is broken by defects which can be impurities, crystallographic point defects or extended defects such as dislocations, or surfaces. As a result, energy levels appear within the band gap and transitions toward these levels correspond to electron or hole trapping.

In the case of photoconductive devices, the selective trapping of one charge carrier (electron or hole) allows the quantum efficiency to reach high values. Let us assume that one hole is efficiently trapped by a defect level (represented by the black symbol on Figure 1b), the electrons will be injected by the device contacts until one of them will be trapped and recombines with the trapped hole. If the electron trapping cross section is small, then a large number of electrons will cross the device before recombination (this electron flow corresponds to the red arrow on Figure 1b), leading to a so‐called photoconductive gain [4, 5] *g* given by the ratio of the electron capture time τ_e_ to the transit time τ_t_.

For short devices and large electric fields, the transit time can be short and the photoconductive gain very large, reaching up to few decades [6, 7]. In polymer‐inorganic composite materials, trapped electrons at interface states assist hole injection and thus enhance gain [8]. Defective layers are sometimes intentionally introduced in high‐quality materials to trap charges and provide a gain [9]. Note that defects can also be intentionally introduced to increase the doping level [10]. In 2D materials trapping by defects also leads to photoconductive gain and a large response [11]. However, the detector performance is often overestimated by ignoring the impact of the gain on the noise [12, 13]. Indeed, the large response due to the photoconductive gain is most of the time associated with a high dark current and a significant noise. So, the detectivity is generally not very high.

In the usual case of photoconductive detectors, the fundamental limitation arises from the large dark current. A typical conductive device is symmetric and an applied electric field is needed to create an electric field (Figure 1b). This electric field remains relatively small, allowing for carrier capture. This potential profile strongly contrasts with that in a photovoltaic device (Figure 1a). Indeed, a strong electric field, without external bias, separates electrons and holes with a negligible carrier capture. Between these two extreme cases, one can define a structure which is by nature asymmetric (as a photodiode) but where a forward bias is applied to reduce the asymmetry (Figure 1c). The remaining internal electric field still generates a photovoltaic response with an electron flow from left to right (corresponding to the blue arrow). On the contrary, the potential at the contacts imposes a dark current related to electrons injected from right to left, and superposed to a possible photoconductive current (represented by the green arrow).

Contrary to the pure photoconductive case (Figure 1b) the dark current in the mixt mode (Figure 1c) is limited by the potential barrier (on the left side of the structure) while the photoconductive current can be large if trapping happens on defect levels (illustrated by the red arrow). Hence, the photoconductive current can be larger than the dark current. The photoconductive (green and red arrows) and the photovoltaic (blue arrow) currents are of opposite sign so the photoconductive current increases with positive bias while the photovoltaic part decreases. Therefore, there will be a positive bias V_0_ at which the global response vanishes. For all the biases smaller than V_0_, including zero bias, the photocurrent is negative. This is the usual operating regime of solar cells and is not the regime we are interested in here. For biases above V_0_ the photocurrent is positive and the dark current drastically increases when the bias reaches the photodiode turn‐on voltage. So, there is a bias regime between V_0_ and the diode turn‐on voltage for which the dark current remains rather low. Combining this low dark current with the large photoconductive response related to the defect trapping phenomenon, a high detectivity is potentially achievable. This is the regime of interest in this paper, and we will call this operation scheme the defect‐mediated detection regime.

Surprisingly, this regime has never been explored in detail. This may be explained by two additional conditions. First, the photodiode must remain rectifying even in the presence of a large density of defects. This turns out to be difficult in materials such as Si or GaAs, due to their moderate band gap energy. On the contrary, GaN devices (LEDs and transistors) have demonstrated extremely high performances [14] in spite of a large density of dislocations (10^8^ cm^−2^). The second condition concerns the carrier injection. Indeed, the electron‐hole pairs have to be injected throughout the entire active region. In addition, relatively thick active regions have to be considered as we will demonstrate that the influence of carrier trapping becomes more pronounced as the thickness increases. Hence, the excitation needs to spatially extend over few microns. Optical excitation in most of direct band gap semiconductors is associated with absorption coefficients in the range of 10^4^ cm^−1^. So, for optical excitation energies above the band gap, the absorption length is around 1 µm and the effect of trapping remains limited. Excitation at smaller energies might be possible to reach longer penetration depth. However, the absorption spectrum varies rapidly around the band gap which makes the optimization difficult. While the mentioned condition is difficult to obtain with optical excitation it can be easily reached with high energy protons. For proton energy of 64 MeV the stopping range in GaN is 8 mm, providing a perfectly homogeneous excitation over large thicknesses. In this study we will explore both theoretically and experimentally our proposal that imperfections may lead to high performance in semiconductor detectors by using GaN excited by high energy protons.

## 2 Results

### 2.1 A Model System for the Defect‐Mediated Detection Regime

As a model material, we use GaN as its band gap energy is large (3.43 eV at 300 K) and defect levels can be created in the band gap, at distant energies from the valence and conduction bands. This enables the combination of efficient carrier trapping with small thermal de‐trapping rates and reduced tunnel transport via hopping processes, thereby preserving the electrical properties of the devices. While the residual doping remains n‐type in GaN, electron trapping on deep levels effectively reduces the net charge carrier concentration below 10^15^ cm^−2^. Consequently, depletion regions above a few µm can be obtained in Schottky or pin diodes. The simplest structure was chosen for the modeling, a Schottky diode, with a limited number of parameters, namely the Schottky barrier height, the residual donor density and the trap density. Irradiation with high‐energy protons yields a homogeneous generation of electron‐hole pairs throughout the entire active region. The electromagnetic interaction between protons and matter is described by the Bethe theory [15] which we experimentally confirmed to be applicable to GaN [16]. The energy deposited in the material at a given position generates electron‐hole pairs. See the methods section for details. Transport is modeled by the drift‐diffusion laws for electrons and holes while carrier capture by traps is described by trapping rates:

(1) $$R_{i}=c_{i}T_{i}\left[i\right]$$

where i = e or h for electrons or holes, T_i_ is the density of traps occupied by an electron and capturing a hole (h), or empty and capturing an electron (e), and c_i_ is the capture coefficient. Since holes exhibit a larger effective mass than electrons, their capture coefficient is accordingly assumed to be higher. Modeling is performed at 300 K so the thermal ionization is negligible as related energies are much larger than kT. Hence the dynamics of traps is dominated by electron and hole captures. The equations are solved iteratively in the time domain and with a spatial discretization. The dynamic of free charge is very fast, with typical times given by the dielectric relaxation time ∈/σ, where ε and σ are the dielectric constant and conductivity, respectively. On the other hand, the trap dynamic is much slower (up to ms or larger). The steady state free‐carrier densities and the trap occupancy are calculated using a dual‐timescale iterative approach to capture both fast and slow dynamics. First, the dark current is deduced from the carrier density (the holes are neglected here) [17]:

(2) $${J_{dark}}_{=}eD_{n}\frac{{\left[n\left(x\right)e^{-eV_{d}\left(x\right)/kT}\right]}_{0}^{W}}{\int_{0}^{W}e^{-eV_{d}\left(x\right)/kT}dx}$$

where D_n_ is the electron diffusion constant, V_d_ is the electric potential in the diode under dark condition, and W is the depletion region thickness. Second, under irradiation, the trap occupancy is modified, leading to changes in both the potential profile, the depletion region thickness and the electron density. The current under irradiation is calculated with the same formulae but with the modified potential and density values. The difference between the current and the dark current defines the photoconductive response (by commodity we keep using the “photo” prefix although we use protons instead of photons). The photoconductive response is positive for a positive bias. In addition, the electrons and holes generated under irradiation are swept by the electric field, leading to a photovoltaic response, that remains negative for all biases up to the Schottky barrier height (taken as 1 eV), where it vanishes. This behavior is the usual response of a defect‐free material. In the presence of defects, it is slightly reduced as will be shown.

Schottky diodes with an active region ranging from 1 to 10 µm were modeled. The residual donor density is set to 10^16^ cm^−3^. The trap density is varied from 10^13^ to 10^16^ cm^−3^, the upper value corresponding to a complete compensation. The proton current I_proton_ is varied from sub pA values to 100 nA. The relevant parameter is the current density, which is defined from the experimental conditions, so that 1 pA corresponds to 1.32 pA/cm^2^ when a single scattering system (see the Experimental Section) is used. The diode area was set to 800 µm × 800 µm, consistent with the experimental conditions.

**FIGURE 2:**
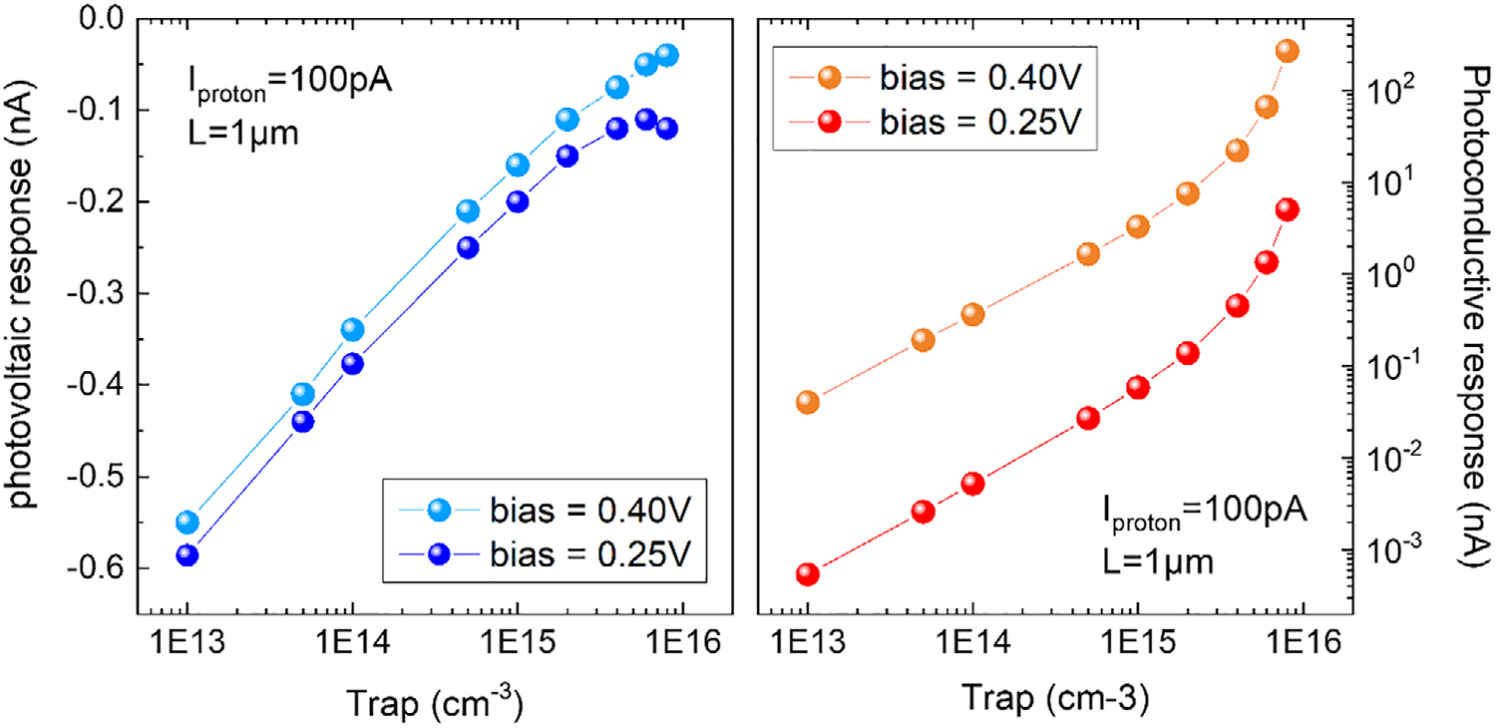
Simulated response of a GaN diode in the defect‐mediated detection regime as a function of the trap density. Photovoltaic (left) and photoconductive (right) responses in a Schottky diode with an active region of 1 µm irradiated by a proton beam (beam current of 100 pA). The photovoltaic response varies as the logarithm of the trap density, while the photoconductive one varies more than linearly with it.

The photovoltaic response of a 1 µm‐thick diode is first evaluated as a function of trap density for a current of 100 pA (Figure 2a) and at two positive biases (0.25 and 0.4 V), both below the turn‐on voltage. The response is negative with a larger absolute value at 0.25 V than at 0.4 V. This behavior is expected, as increasing the positive bias reduces the depletion region thickness and weakens the internal electric field, thereby diminishing the photovoltaic response. As the trap density increases, the photovoltaic response decreases (in absolute value) as more and more electrons and holes recombine on traps. We note that this decrease is rather slow, with an approximately logarithmic dependence on trap density.

Subsequently, we evaluated the photoconductive response (Figure 2b) to further investigate the impact of trap states. The response is positive and strongly increases with the positive bias. This is due to the exponential activation of the current over the energy barrier, which linearly decreases with positive bias. The most important point is the pronounced enhancement of the photoconductive response with the trap density. It is clear that this response vanishes in the absence of traps. This crucial aspect has already been noted by many authors in conventional photoconductors [5, 18]. When the trap density reaches the residual donor density, the photoconductive response increases strongly, showing a divergence behavior. The photoconductive response surpasses the photovoltaic one (in absolute value) for a trap density and/or a positive bias sufficiently large. Hence, the typical negative response observed at small positive biases in high‐quality Si solar cells may be replaced by a positive one in a defective material, due to the photoconductive contribution.

**FIGURE 3:**
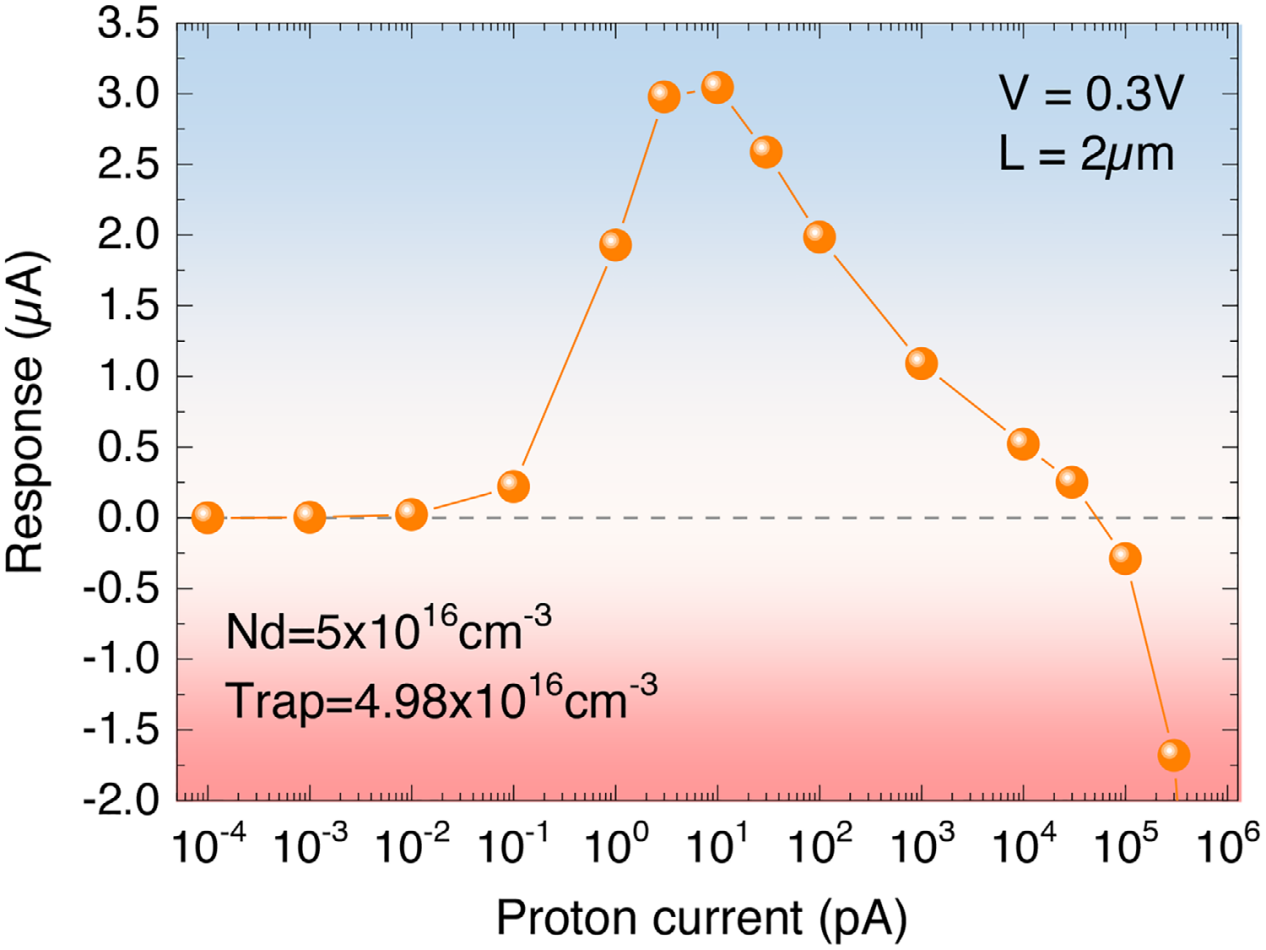
Simulated response of a GaN diode in the defect‐mediated detection regime as a function of the incident proton current. Total response in a Schottky diode with an active region of 2 µm at V = 0.3 V, as a function of the proton current. The donor density is 5×10^16^ cm^−3^ and the trap density is 4.98×10^16^ cm^−3^. The signal changes sign for a proton current of 5×10^4^ pA. The response is dominated by the photoconductive response at low proton current, and by the photovoltaic one at high current.

We now study how both responses evolve with the proton current. The trap density (4.98×10^16^ cm^−3^) is chosen to be very close to the donor density (5×10^16^ cm^−3^) to reach a large compensation. First, the photovoltaic response exhibits an almost linear dependance on the proton current across the entire range, as expected. In contrast, the photoconductive response increases linearly with proton current up to approximately 1pA, beyond which it saturates and even slightly decreases at higher currents. The evolution of the trap occupancy provides insight into this behavior. In the dark and at very low proton currents, electrons transfer from shallow donor states to traps, effectively reducing the doping density. As a result, the depletion region is large. At higher proton currents, irradiation‐induced holes are trapped and subsequently recombine with electrons, leading to neutralization of the traps. This increases the effective doping density and causes the depletion region to shrink. The resulting reconfiguration of the band profile gives rise to the photoconductive response. However, this process saturates around a proton current of 1pA, when all traps are neutral. Beyond this point, the photoconductive response does not increase anymore. As a consequence, the total response is dominated by the photoconductive contribution at low proton currents, resulting in a net positive signal, while at higher proton currents, the photovoltaic component prevails, leading to a net negative response. Hence, the total response changes sign as the proton current increases (Figure 3), a rather unusual behavior. Conversely, the system exhibits high sensitivity at very low proton currents, a feature we aim to exploit.

Let us briefly comment the choice of trap density made in Figure 3. When the trap density is chosen to be larger than the donor density (overcompensation), most electrons remain trapped even under proton irradiation at small and moderate proton currents and the depletion region remains constant, equal to the undoped layer thickness. The photoconductive response is negligible. A very large proton current is needed to significantly empty the traps and the same behavior as the one in Figure 3 is observed but at a higher proton current. Conversely, when the trap density is much smaller than the donor density, the same effect as the one shown in Figure 3 can be observed. The photoconductive response is however smaller and the change of sign arises at a smaller proton current (see Figure S1). Hence the trade‐off (largest response and smallest proton current at the change of sign) is obtained at a trap density close to the donor density, as in Figure 3.

**FIGURE 4:**
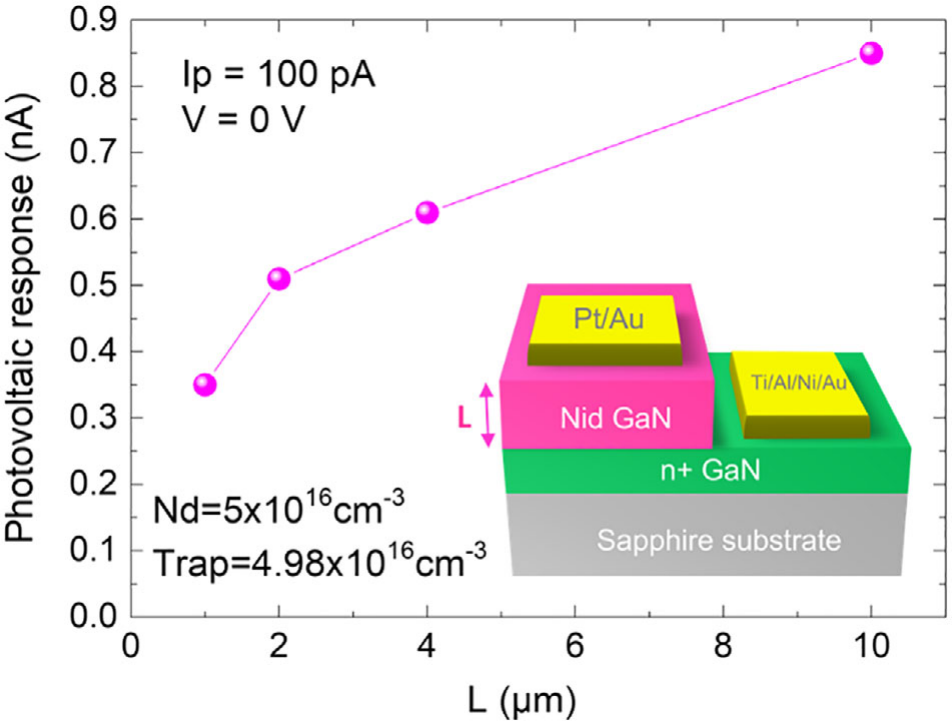
Simulated response in pure photovoltaic mode as a function of the active region thickness. Response (in absolute value) in a Schottky diode at 0 V under irradiation at a proton current of 100 pA, as a function of the active region thickness. The material is almost fully compensated. The response departs from the linear variation which could be expected if dynamic trapping effects would be neglected.

The evolution of trap occupancy under irradiation is not restricted to positive bias conditions. We investigated this behavior at 0 V and observed similar hole trapping and a corresponding increase in the effective doping with increasing proton current. Interestingly, at 0 V, no photoconductive response is observed, and the total signal is solely governed by the photovoltaic contribution. We calculated this response as a function of the active region thickness L in the case of an almost fully compensated material (Figure 4). In this regime, the active region is fully depleted by the internal field at 0 V in the dark. Since the photovoltaic response increases linearly with the thickness of the depleted region, a linear dependence of the response on L is expected. However, this expected linear trend is not observed in Figure 4, where the increase is clearly sublinear. This deviation arises from the fact that, under irradiation, the effective doping increases as traps become progressively emptied. As a result, the depletion region no longer extends across the entire active region.

### 2.2 Experimental Defect‐Mediated Detection Regime Demonstrated in GaN Diodes Under Proton Beams

Schottky diodes based on GaN were fabricated (see Experimental Section) with active region thicknesses ranging from 1 to 10 µm. Capacitance measurements revealed that, in the dark at 0 V, the depletion region extends across the entire active region [19], indicating a low effective doping level. Consequently, electron‐capturing defects exist in the material prior to irradiation. The measurements of the diode currents in the dark and under irradiation, which will be presented later, did not change over time or with irradiation, indicating that the effect of defects that could be created by irradiation is negligible. An effect of irradiation on the diode properties could only be observed at doses 1000 times higher than those used in this experiment. Measurements under proton irradiation were then conducted at the Centre Antoine Lacassagne using high‐energy protons of 64 MeV (see Experimental Section).

The response at 0 V was first measured on various diodes. The measured values exhibited a dispersion of approximately 30% among nominally identical detectors. Figure S2 shows that the response does not follow the linear increase with active region thickness, as predicted by the theoretical model, highlighting the crucial role of traps and hole capture processes.

We next measured the response as a function of bias under small positive voltages (corresponding to the defect‐mediated detection regime) using a proton current of 100 pA. For all diodes, regardless of active region thickness, the response exponentially increases with applied bias. As the dark current follows a similar exponential dependence, it is instructive to consider the ratio of the response to the dark current. This ratio varies from diode to diode, and from sample to sample. However, in most cases, it significantly exceeds unity, often reaching several orders of magnitude, thereby demonstrating a very high sensitivity. This ratio is maximum at a bias that varies from sample to sample, typically within the range of 0.35–0.6 V (Figure S3).

In this bias range, we studied the dependence of the response on the proton current (Figure S4). The response saturates at high proton current, for currents larger than a few hundreds of pA. As the bias is rather large (more than one half the turn on voltage of 0.8 V), the saturation is not complete and the change of sign not observed. Moving to a pin diode and a bias of 0.6 V much smaller than the turn on voltage of 3 V, we could observe that the response changes sign when the proton current increases, from positive at low current to negative at large current (see Figure S5). The large variations of behavior among diodes can be explained by the level of compensation of the doping, which varies from wafer to wafer, and even on a given wafer, varies from the center to the edge due small variations of growth temperature, V/III ratio.

Hence, all theoretical predictions were experimentally confirmed, including some rather unusual effects (for instance the inversion of the signal sign with increasing incident irradiation level) which, to the best of our knowledge, have not been previously observed.

### 2.3 Toward an Unprecedented Sensitivity of a Semiconductor Diode in a Proton Beam

**FIGURE 5:**
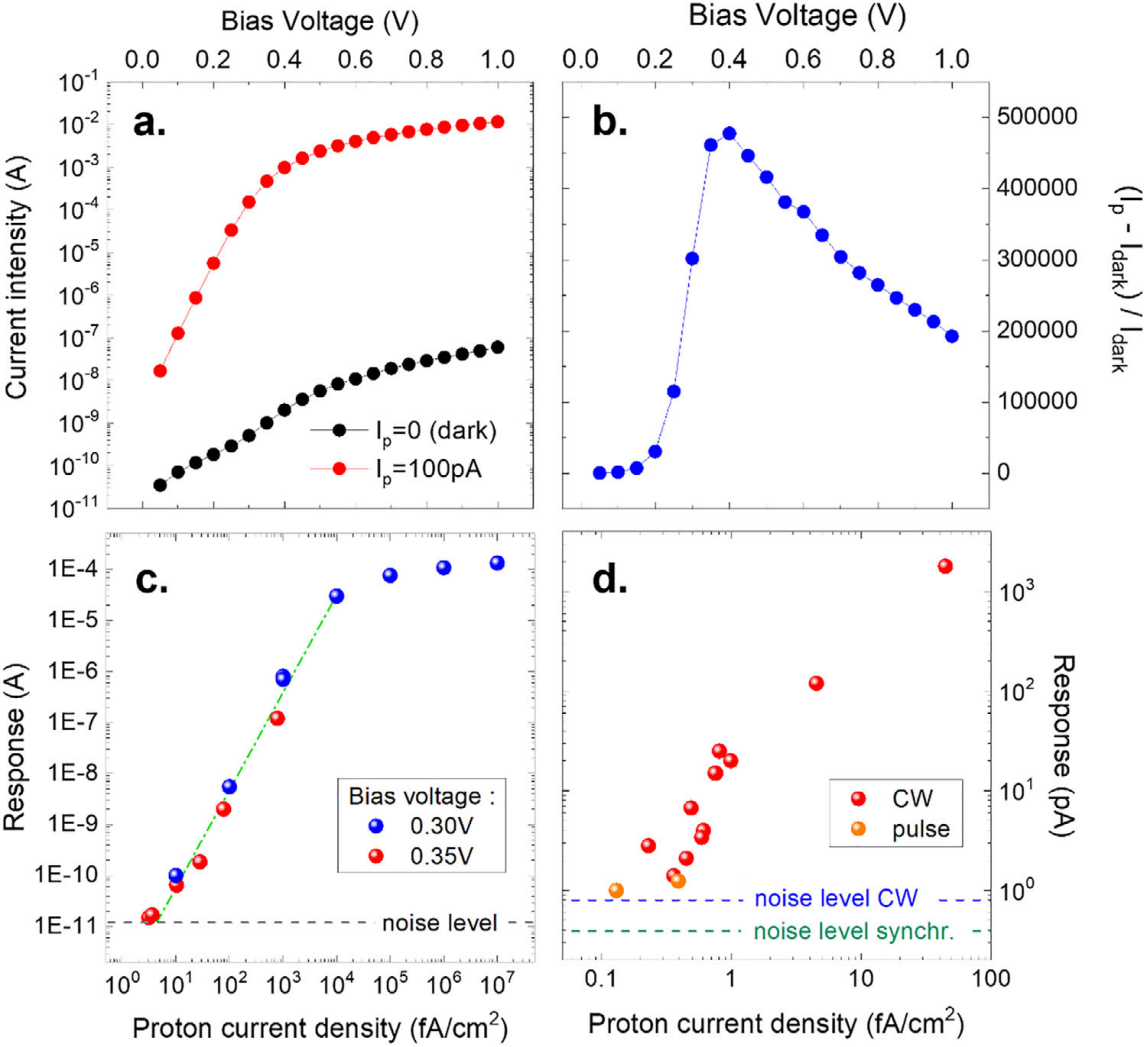
Experimental response of a Schottky diode in a proton beam. (a) Dark current and response to protons of a Schottky diode with a 4 µm active region. (b) Ratio of response to dark current, for a proton current of 100 pA. The ratio reaches high values, indicative of pronounced sensitivity that will be exploited in (c, d). (c) Measurements with a Gaussian beam at two different biases. The integration time is 20 ms. (d) Measurements at 0.35 V with a broad beam allowing to reach lower proton current densities in CW and in pulse mode.

A Schottky diode with a 4 µm active region was measured at a proton current of 100 pA. The response, dominated by the positive photoconductive response is several decades larger than the dark current (Figure 5a). The ratio of response to dark current (Figure 5b) reaches a value close to 5×10^5^ at a bias of 0.35–0.4 V

This is the highest ratio between the dark current and the current under proton irradiation observed. Notably, this occurs at a bias voltage where the absolute dark current, and consequently the associated electronic noise, remain low. At a bias of 0.35 V, the distribution of dark currents has a standard deviation of 12 pA with an apparatus integration time of 20 ms. This standard deviation sets the detection threshold, corresponding to a minimum detectable current for a bandwidth of 50 Hz. The diode response was measured at bias voltages of 0.3 and 0.35 V as a function of the proton current density down to extremely low flux levels. The response decreases with current density at a rate slightly exceeding linearity (Figure 5c). At higher proton fluxes, the response exhibits a saturation, in agreement with the predictions of our model. Having the response almost identical at 0.3 and 0.35 V indicates that theses biases are close to the optimum voltage, where the proton to dark current ratio reaches its maximum. Measurements at 0.4 V confirm that the sensitivity is reduced at higher bias and at low proton currents. Accurate measurements become challenging for densities lower than 5 fA/cm^2^ as the signal is weak and quantifying the total proton current becomes difficult. To achieve even lower proton current densities, a single‐scattering configuration incorporating a 550 µm‐thick tantalum diffuser was used to produce a broad beam (see Experimental Section). After careful beam calibrations proton current densities as low as 0.1 fA/cm^2^ were reliably achieved. The diode current measurement setup was also modified by introducing an ultra‐low noise current amplifier (Femto DDPCA‐300) with a gain set to 10^8^ V/A and a 0.2 Hz low‐pass filter, well suited to the slow time response of the diode in this regime. As shown in Figure 5d, the noise level (estimated from the dispersion of the response signal) is reduced to approximately 1 pA enabling reliable measurements down to about 2 pA of signal in continuous mode.

**FIGURE 6:**
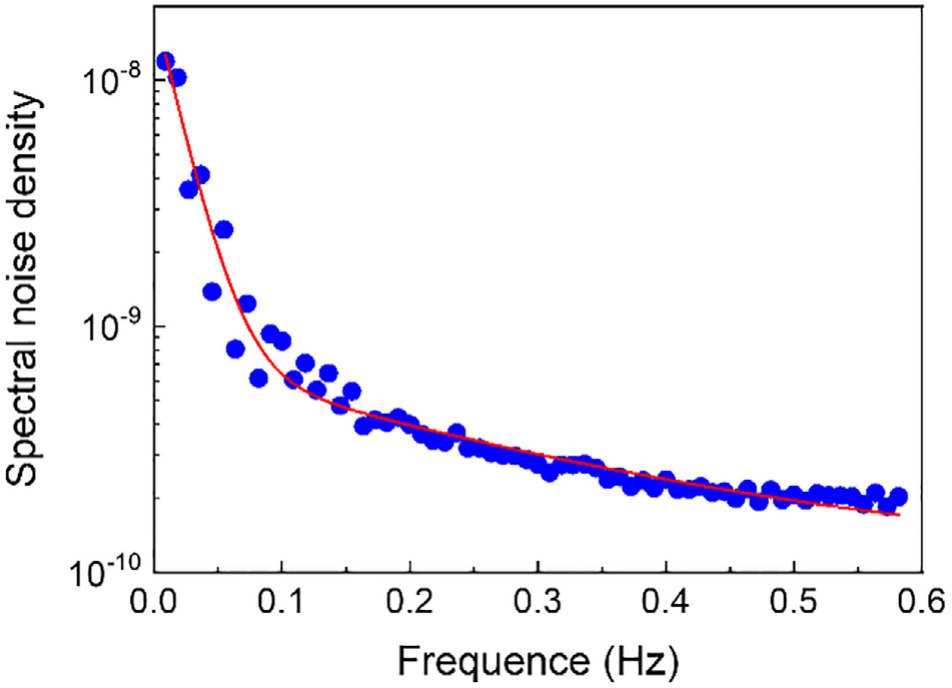
Analysis of the noise in the Shottky diode working in the defect‐mediated detection regime. Spectral density of noise measured on the voltage signal delivered by the current trans‐amplifier of gain 10^8^ V/A.

To gain deeper insight into the noise spectrum, the signal was recorded over a 200 s interval, and its autocorrelation function was calculated from:

(3) $$AC\left(n\right)=1/N\sum_{i=1}^{N}f\left(i\right)f\left(i+n\right)$$

where i denotes the ith signal value and N is the total number of data points (N = 60, corresponding to a 50 s segment). The spectral noise density was then obtained via Fourier transformation of the autocorrelation function and is presented in Figure 6.

The spectral noise density decreases with frequency before reaching a constant value (white noise) for frequencies above 0.5 Hz. An excess noise component is noticeable below 0.2 Hz, partly attributable to the frequency filter of the amplifier. It is worth noting that the autocorrelation function was evaluated over a 50 s interval, imposing a lower bound of 0.02 Hz for the noise. From the spectral noise density of 2×10^−10^ V^2^ we extract a voltage noise of 1.45×10^−5 ^V, which, given the amplifier gain of 10^8 ^V/A, corresponds to a current noise of 1.45×10^−13^ A. This estimated value is slightly lower than the current dispersion as it does not include the lower‐frequency noise. This spectral noise analysis suggests that improved measurement sensitivity could be achieved by operating at a non‐zero frequency, while remaining at frequencies compatible with the inverse of the response time, which is over 10 s. Therefore, the temporal profile of the proton beam was modified to a pulse mode with a 20 s period (10s ON/10s OFF). Figure S6 shows the time‐resolved signal. Due to the diode response time, the response induced by the square proton pulse can be approximated by a *A*cos(ωt) variation, where 2*A* corresponds to the full response amplitude. To extract *A*, a numerical lock‐in method was employed: the signal was multiplied by a cosine function of the same frequency, the relative phase was adjusted, and the result was averaged over time. At the lowest investigated proton current density of 0.13 fA/cm^2^, a diode response of 1 pA was measured (orange circle on Figure 5d). Given the diode's active area, this corresponds to about 5 protons/s on the diode. The noise level was calculated by subtracting the fitted cosine function from the measured signal and estimating the standard deviation of the residuals. The noise level, estimated at approximately 0.4 pA, is lower than that observed in continuous mode operation, owing to the suppression of low‐frequency noise components (below 0.05 Hz) in the pulsed mode. Hence, the extrapolated lowest proton current density measurable for a signal to noise ratio of unity corresponds to 2 proton per second per diode. Increasing the integration time from 0.7 to 2.8 s (unfortunately not currently accessible with our equipment) would allow gaining an additional factor of 2, potentially enabling the detection of a single proton per second per diode. The main limitation in our measurements lies in the ability to accurately control and monitor such a low proton current density, underscoring the need of a lower‐current monitoring system. To our knowledge, this level of sensitivity has not previously been achieved with semiconductor detectors for high energy protons. While higher sensitivities ‐ extending to the quantum level detection – have been demonstrated with avalanche photodiodes and perovskites‐based structures for photon detection [20, 21] and for X‐ray detection [22], similar performance for high‐energy proton detection has only recently been approached in perovskite‐based scintillators [23]. From the measured response, we extract the photoconductive gain *g*. At a proton current density of 45 fA/cm^2^, the diode yields a response of 1800 pA. At 54.7 MeV protons, the SRIM software indicates an energy deposition of 4.82 keV per µm in GaN, corresponding to a total of 19.3 keV deposited within the diode's active layer. Applying the empirical rule proposed by van Roosbroeck [24] and Klein [25], which states that approximately three times the semiconductor bandgap energy is required to generate a single electron–hole pair under high‐energy excitation, we estimate a charge generation efficiency of C = 1875 carriers per incident proton. The proton flux reaching the diode is given by: ϕ = 45 × 10^−15^/*e* × *S* where *S* is the diode surface.

The diode response is given by *R* = ϕ × *C* × *g* × *e*, which yields a photoconductive gain *g* = 3.3×10^3^.

This gain can be used to calculate the noise current given by $i_{noise}=\sqrt{4egI\Delta \nu }$.

The integration time of 0.7 s corresponds to Δν = 1,3 Hz. At a bias voltage of 0.35 V, the measured dark current is 26 pA. The corresponding theoretical noise current is calculated to be 0.27 pA, in reasonable agreement with the experimentally observed current dispersion of 0.4 pA when accounting for additional noise contributions due to the environment and measurement apparatus.

## 3 Discussion

We would like in this short section to discuss the impact and limits of this defect‐mediated detection regime for device applications. At a bias of 0.35 V, corresponding to the defect‐mediated detection regime, the detector exhibits a notably slow response, with time constants exceeding 10 s and showing no significant dependence on the incident proton current density. This behavior is attributed to trap dynamics, particularly the electron trapping time. The combination of long response time and large signal response reflects the conservation of the response‐bandwidth product. Nevertheless, for applications where long response times are acceptable, operation in this defect‐mediated detection regime enables exceptionally high sensitivity. Conventional photoconductors based on homogeneous defective materials also exhibit large photoresponses, however they typically suffer from high dark current and a large noise current, which limits their overall detectivity. Their response time is moderately long (typically in the millisecond to second range), corresponding to a response‐bandwidth product trade‐off different from the one in the defect‐mediated detection regime discussed here. The Schottky diodes investigated here can also operate in the conventional photoconductive regime, under external bias exceeding their built‐in potential (∼1 V). The sensitivity is however hindered by the strong dark current. Under reverse bias, or at zero bias, the devices exhibit an almost ideal photovoltaic response with quantum efficiency approaching unity and a response time below the 20 ms resolution limit of our setup. In this regime, proton current densities as low as 0.25 pA/cm^2^ can be detected. The defect‐mediated detection scheme allows an enhancement of the sensitivity by three orders of magnitude (reaching 0.13 fA/cm^2^). Increasing the defect density is expected to amplify this phenomenon. However, there is a limit to this beneficial effect. When the materials become too defective, the Schottky barrier becomes more transparent to electrons due to trap‐assisted tunneling, leading to a loss of the diode rectifying behavior, a rise in dark current, and a degradation of the sensitivity. Defects densities on the order of 10^18^ cm^−3^ correspond to an average spacing of 10 nm between defects, enabling large tunnel currents. In contrast, densities around 10^16^ cm^−3^ likely represent an optimal value for this defect‐mediated detection regime in GaN.

## 4 Conclusion

We identify the defect‐mediated detection regime as an extreme manifestation of the bandwidth–sensitivity trade‐off. In this regime, Schottky and p–i–n diodes operated under forward bias, yet below the turn‐on voltage, simultaneously exhibit low dark currents and high photoconductive gain. All features predicted by simulation were experimentally confirmed in GaN diodes exposed to proton irradiation. Although previously overlooked, this regime is likely to occur in any semiconductor exhibiting strong doping compensation, and under excitation by both light and high‐energy particles.

In the optical domain, such conditions may arise near the band‐edge, where absorption coefficients lie between 0.1 and 1 µm^−1^ over a narrow spectral range. Conversely, X‐ray interactions naturally yield similarly low absorption coefficients. A key trade‐off, however, is the slow response time, typically on the order of several seconds. Despite this limitation, the combination of high sensitivity and prolonged integration time can be advantageous for imaging and low flux particule detection. A particularly compelling application lies in the context of proton therapy, where precise imaging is required prior to treatment in order to accurately model tissue interactions and calibrate dose delivery. At present, this step is typically performed using X‐ray imaging, and proton‐equivalent tissue properties are inferred through indirect calculations, introducing uncertainties into the treatment planning process. Direct imaging with the same therapeutic proton beam would eliminate this source of error. Realizing such a capability requires detectors that combine high sensitivity and fine spatial resolution. The defect‐mediated detection regime demonstrated here satisfies these requirements, offering a promising platform for proton imaging in clinical workflows, where response times of several seconds remain compatible with procedural constraints. CT scans (X‐ray patient imaging) that are performed prior to proton irradiation to tune the dose planning are from 10 s to 1 min long. Meanwhile, the patient must remain still. Therefore, it is possible to consider replacing the CT scan with proton imaging, with time scales on the order of 10 s.

Compared to conventional detectors such as scintillators, direct‐readout semiconductor devices offer improved spatial resolution, enabled by micron‐scale pixel pitches. Moreover, despite the possibility of saturation at high excitation levels, the dynamic range in our GaN diodes remains superior, providing an additional advantage for applications in X‐ray and high‐energy particle detection.

## 5 Experimental Section

### 5.1 Model

The electron and hole created by the proton beam is calculated from the power deposited at a position *x* of the diode which is calculated from the SRIM software (https://www.srim.org). The power δ*P* is converted into electron and hole generation rate by the usual rule (Klein's model) [25]:

$$G=\frac{\delta P}{3E_{G}}$$

The electron current is calculated from the following equations:

$$J_{n}=-n\mu_{n}F-D_{n}\frac{\partial n}{\partial x}$$

$$\frac{\partial n}{\partial t}=G-R_{n}-\frac{\partial J_{n}}{\partial x}$$

And the same for holes.

The band profile is calculated from the Poisson equation:

$$\Delta V+\frac{\rho }{\varepsilon }=0$$

where the charge density ρ includes the electron, hole, ionized donor and charged trap densities.

Trap dynamics is described by:

$$\frac{\partial n}{\partial t}=G-R_{n}-\frac{\partial J_{n}}{\partial x}$$

where T is the neutral trap density which can trap an electron and T‐ is density of traps charged with an electron and which can trap a hole. Thermal emission is neglected as energies are much larger than kT.

Equations are solved in the time domain iteratively. As the trap dynamics is much slower than the free charge dynamics, a two‐step procedure is used to minimize the calculation time.

### 5.2 Sample Preparation

Diodes were fabricated on GaN layers. GaN layers were grown by metal organic vapor phase epitaxy on sapphire substrates. A 2 µm thick non‐intentionally doped GaN layer was grown first including a 3D growth mode to reduce the dislocation density in the range of a few 10^8^ cm^−2^. Then a 2 µm GaN layer was grown with a n‐doping of 10^18^ cm^−3^. Finally, the non‐intentionally doped GaN active region was grown with a thickness between 1 and 10 µm. Growth conditions were chosen to minimize the residual carrier density by compensating the residual donors (silicon and oxygen) by electron traps.

Diodes were then processed in a clean room. Mesa was defined by optical lithography and etched by ECR reactive ion etching down to the bottom contact layer. A TiAl ohmic contact was deposited on the n‐type GaN layer, and annealed at 750°C for 4 min. The mask included various diode sizes: we used in this study the 800 µm size diodes. Then large Schottky contacts were deposited on the top of the mesa, based on 10 nm of Pt followed by 100 nm of Au. A thick metal layer was finally deposited on the contacts for facilitating the wire bonding.

Samples with many diodes were mounted on ceramic chips and diodes were wire bonded to external electrical connections. Chips are mounted on a x‐y translation stage so that they can be moved and aligned in the proton beam.

All measurements were made at room temperature. Electrical measurements were performed with a Keithley 2410‐C Source‐meter, with an apparatus integration time of 20 or 700 ms.

### 5.3 Proton Beam

MEDICYC is a 65 MeV isochronous cyclotron hosted by the Centre Antoine Lacassagne in Nice, France. It feeds two beamlines: a primary clinical beamline dedicated to ocular melanoma treatment using proton therapy [26], and a secondary research and development (R&D) beamline for experimental studies [27].

The R&D beamline was equipped with both single and double scattering systems, capable of producing either a Gaussian beam profile or a flat beam profile (50 or 100 mm in diameter) at the position of the device under test (DUT). For this study, the Gaussian beam was selected using the single scattering system with the highest scattering angle, as it provides the lowest achievable beam flux. Considering the propagation of proton in air and through the line window, we can estimate the proton energy incident on the detector to be 63.5 MeV.

To minimize the beam flux, a 550 µm tantalum foil was used as a scattering medium, and the DUT was placed 3 m downstream from this foil. This configuration increased beam diffusion while minimizing energy loss due to the scattering material. Considering the additional propagation of proton in air and through the tantalum foil, we can estimate the proton energy incident on the detector to be 54.7 MeV. The energy deposited in the GaN detector is barely affected by this energy change: 4.82 keV/µm versus 4.31 keV/µm at 54.7 MeV versus 63.5 MeV respectively (SRIM).

Beam monitoring was carried out using a large‐diameter transmission ionization chamber (IC) positioned just upstream of the scattering foil. As such, all particles pass through the IC before being diffused. The IC provided a flux measurement before any significant scattering occurs (at a few centimeters from the foil), while a Faraday Cup (FC) located 3 m away—at the DUT position—measures the final current. A 10 cm diameter collimator was placed directly in front of the FC. Beam profile measurements were performed using the LYNX detector from IBA, a pixelated scintillator system with an effective spatial resolution of 0.5 mm. These measurements revealed that the intensity drop from the center of the Gaussian profile to a point 5 cm off‐axis was approximately 4%. Therefore, to ensure consistency, all DUTs were positioned precisely at the beam center using a high‐precision laser alignment system.

**FIGURE 7:**
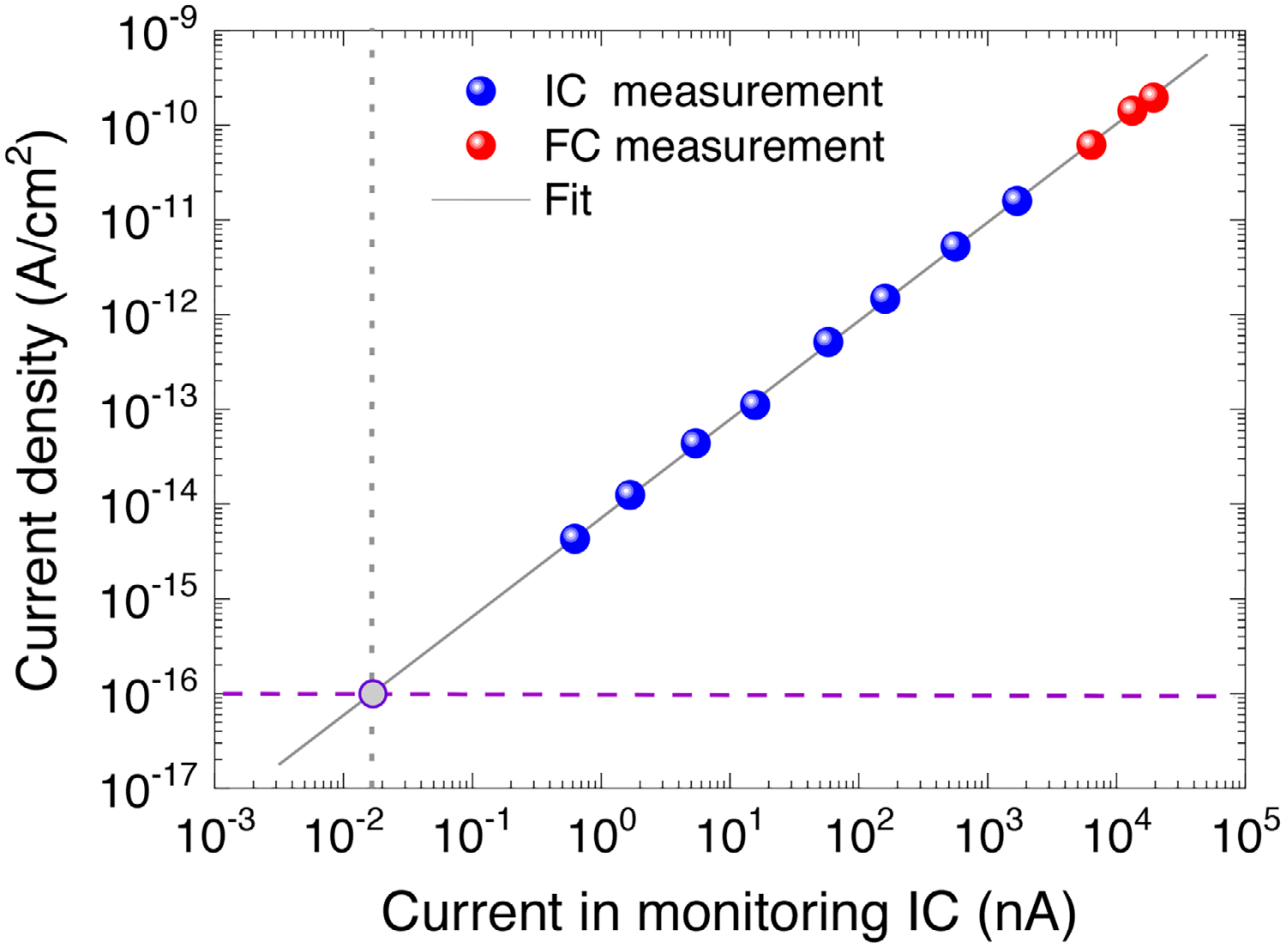
Calibration of the proton current based on an ionization chamber and a Faraday cup. Current density at the DUT position as a function of the current measured in the monitoring ionization chamber. The current density was determined using a Faraday cup, a secondary ionization chamber, and is also illustrated based on Monte Carlo simulations.

By comparing the mean current density measured by the FC (i.e., current divided by collection surface) to the current measured by the IC, we can establish a calibration curve linking the IC reading to the actual proton flux at the DUT position. Since we do not apply any correction for the beam inhomogeneity, the resulting flux estimation at the DUT position is accurate to within a few percent. This initial estimation is shown as red dots in the graph below. See Figure 7.

However, because the Faraday Cup detects individual protons without signal amplification, measurements must be conducted at relatively high beam currents (above the nanoampere range), which is still high compared to what is ideally needed (around 0.1 pA). The required current level (0.1fA/cm^2^) is indicated by the horizontal purple line in the graph.

To achieve lower fluxes and obtain a broader range of measurement points, the FC is replaced with a second ionization chamber with a gain factor of 130, coupled with a more sensitive electrometer. This enables a second set of measurements (represented by blue points). This second IC was chosen as the calibration reference, as it allowed for a greater number of data points closer to the target current range. Proton current densities close to the target point were estimated from extrapolation of the calibration curve shown in Figure 7.

### 5.4 Measurements

Diodes were measured with and without proton irradiation. The response was precisely measured by switching the beam on and off and by time recording the diode current at a given bias. The magnitude of the step gives the response value and the transient gives the response time. In the pulse mode, the beam was turned ON and OFF every 10 s, corresponding to a period T = 20 s. The signal was measured as a function of time. We assume that the signal varies as Acos(ωt) with ω equal = 2π/T. To calculate A, we use a numerical lock‐in method. We multiply the signal by a cos function of the same frequency, we adjust the relative phase between both, and average over time. The mean value is equal to A/2, and the response is then calculated to be the peak‐to‐peak variation of the signal, which is thus equal to 2A.

## Author Contributions

J.Y.D. conceived and designed the study, conducted the main experiments, and drafted the paper with contributions from all authors. M.S. was responsible for sample fabrication. E.F. was in charge in epitaxial growth. S.C. supervised clean room processes. P.H., S.M. and M.V. were in charge of operating the proton line facility. M.H. conducted experiment and co‐wrote the paper. All authors reviewed and approved the final paper for submission.

## Conflicts of Interest

The authors declare no conflicts of interest

## Supporting information




**Supporting file**: advs73454‐sup‐0001‐SuppMat.docx.

## Data Availability

The data that support the findings of this study are available from the corresponding author upon reasonable request.
