# Supplementary material for: Imperfection in Semiconductors Leading to High Performance Devices
Source: Adv Sci (Weinh). 2025 Dec 19;13(13):e16270. doi: 10.1002/advs.202516270 (PMC12955859; doi:10.1002/advs.202516270)
Supplement: Supplementary file 1 — Supporting file: advs73454‐sup‐0001‐SuppMat.docx. [file ADVS-13-e16270-s001.docx]

Supplementary information


$$R_{p}=pc_{p}T$$

$$R_{n}=nc_{n}T_{+}$$

**Fig. S1:** **Simulated response of a Schottky diode in a lightly compensated material**. Total response in a Schottky diode with an active region of 2µm at V=0.3V, as a function of the proton current. The donor density is 5×10^16^ cm^-3^ and the trap density is 1×10^16^ cm^-3^. The signal changes sign for a proton current of 300 pA.

**Fig. S2: Experimental response of GaN diodes in the photovoltaic regime.** Response at 0V of Schottky diodes as a function of the active region thickness under a 100 pA proton beam. The response clearly departs from a linear variation with active region thickness.

**Fig. S3 :** **Experimental response to dark current ratio of various GaN diodes in a proton beam.** Response to a proton current of 100 pA of four Schottky diodes with an active region of 10 µm.

**Fig. S4:** **Experimental response of a Schottky diode in the defect-mediated detection regime.** The response of a Schottky diode with an active region of 10 µm saturates when the proton current increases. The bias is too large to observe a decrease of the signal at high proton current. This is better observed in Fig.S5.

**Fig. S5:** **Experimental response of a GaN pin diode in the defect-mediated detection regime.** The response of two pin diodes with an active region of 10µm changes sign at large proton currents. Note that the bias used to reach the defect-mediated detection regime for a pin (0.6V here) has to be larger than for a Schottky diode, according to a larger turn on voltage (3 V for a pin, versus 0.8V for a Schottky diode).

**Fig. S6 :** **Experimental response of a GaN Schottky diode under a pulsed protn beam.** Response of a Schottky diode at a bias of +0.35 V to a proton beam with a density of 0.39 A/cm2. The beam is in pulse mode with a 20 s period. The signal is fitted by a cosinus function. The amplifier gain is 10^8^ V/A.
